# Retraction Note: Comparison of the sagittal profiles among thoracic idiopathic scoliosis patients with different Cobb angles and growth potentials

**DOI:** 10.1186/s13018-015-0181-0

**Published:** 2015-03-26

**Authors:** Bo Ran, Guo-you Zhang, Feng Shen, Jia-yu Chen, Ji-bin Wu, Feng-chao Zhao, Kai-jin Guo, Dun-yi Qi, Bing Zhou, Xiang-yang Chen, Xin-zhu Zhang, Yue-hua Qiao, Ming Li

**Affiliations:** Department of Orthopedics, The Affiliated Hospital of Xuzhou Medical College, No. 99 Huaihai road, Xuzhou, Jiangsu 221006 China; Department of Orthopedics, Changhai Hospital Affiliated to Second Military Medical University, Shanghai, 200433 China; Department of Orthopedics, Kunming General Hospital of Chengdu Military Command, Kunming, 650032 China; Department of Anesthesiology, The Affiliated Hospital of Xuzhou Medical College, No. 99 Huaihai road, Xuzhou, Jiangsu 221006 China; Department of Otolaryngology, The Affiliated Hospital of Xuzhou Medical College, No. 99 Huaihai road, Xuzhou, Jiangsu 221006 China; Institute of Audiology and Speech Science, The Affiliated Hospital of Xuzhou Medical College, Xuzhou, Jiangsu 221006 China

## Retraction

The Publisher and Editor regretfully retract this article [1] because the peer-review process was inappropriately influenced and compromised. As a result, the scientific integrity of the article cannot be guaranteed. A systematic and detailed investigation suggests that a third party was involved in supplying fabricated details of potential peer reviewers for a large number of manuscripts submitted to different journals. In accordance with recommendations from COPE we have retracted all affected published articles, including this one. It was not possible to determine beyond doubt that the authors of this particular article were aware of any third party attempts to manipulate peer review of their manuscript.
